# Evidence of a metabolic reserve in the skeletal muscle of elderly people

**DOI:** 10.18632/aging.101079

**Published:** 2016-11-06

**Authors:** Gwenael Layec, Joel D. Trinity, Corey R. Hart, Yann Le Fur, Jacob R. Sorensen, Eun-Kee Jeong, Russell S. Richardson

**Affiliations:** ^1^ Department of Medicine, Division of Geriatrics, University of Utah, Salt Lake City, UT 84112, USA; ^2^ Geriatric Research, Education, and Clinical Center, George E. Whalen VA Medical Center, Salt Lake City, UT 84112, USA; ^3^ Department of Exercise and Sport Science, University of Utah, Salt Lake City, UT 84112, USA; ^4^ Department of Radiology and Utah Center for Advanced Imaging Research, University of Utah, Salt Lake City, UT 84112, USA; ^5^ CRMBM, Aix-Marseille Universite, CNRS 7339, Marseille, France

**Keywords:** ^31^P-MRS, PCr recovery kinetics, O_2_ availability, mitochondrial ATP synthesis, aging

## Abstract

The purpose of the present study was to determine whether mitochondrial function is limited by O_2_ availability or the intrinsic capacity of mitochondria to synthesize ATP in elderly individuals. To this aim, we examined, in comparison to free-flow conditions (FF), the effect of superimposing reactive hyperemia (RH), induced by a period of brief ischemia during the last min of exercise, on O_2_ availability and mitochondrial function in the calf muscle. 12 healthy, untrained, elderly subjects performed dynamic plantar flexion exercise and phosphorus magnetic resonance spectroscopy (^31^P-MRS), near-infrared spectroscopy (NIRS), and Doppler ultrasound were used to assess muscle metabolism and peripheral hemodynamics. Limb blood flow [area under the curve (AUC), FF: 1.5±0.5L; RH: 3.2±1.1L, P<0.01] and convective O_2_ delivery (AUC, FF: 0.30±0.13L; RH: 0.64±0.29L, P<0.01) were significantly increased in RH in comparison to FF. RH was also associated with significantly higher capillary blood flow (P<0.05) and this resulted in a 33% increase in estimated peak mitochondrial ATP synthesis rate (FF: 24±11 mM.min^−1^; RH: 31±7 mM.min^−1^, P<0.05). These results document a hemodynamic reserve in the contracting calf muscle of the elderly accessible by superimposing reactive hyperemia. Furthermore, this increase in O_2_ availability enhanced mitochondrial function thus indicating a skeletal muscle metabolic reserve despite advancing age and low level of physical activity.

## INTRODUCTION

A hallmark of advancing age is a progressive decline in maximal O_2_ consumption during whole-body exercise that appears to be concomitant to decrements in various central and peripheral components of O_2_ transport to skeletal muscle [1–3]. However, to date, owing to the confounding effects of physical activity and the technical challenges associated with assessing both peripheral O_2_ delivery and mitochondrial function in skeletal muscle *in vivo*, it is still equivocal whether the age-related decrease in O_2_ transport capacity is accompanied by a proportional decline in mitochondrial ATP synthesis capacity [4–10].

The acute manipulation of O_2_ availability combined with the *in vivo* measurement of peak mitochondrial ATP synthesis rate by ^31^P-MRS offers an opportunity to assess the matching of O_2_ supply and demand. Using this approach, it has recently been documented that enhanced O_2_ availability to the muscle, induced by higher fractions of inspired O_2_ (FiO_2_ = 1.0), resulted in unaltered mitochondrial function in the calf muscle of elderly individuals [11]. Somewhat in contrast to this finding, Wray *et al*. [12] reported that enhanced tissue perfusion induced by acute antioxidant administration in elderly subjects improved mitochondrial function, both assessed by MR imaging and spectroscopy techniques, suggesting that a relative hypoperfusion potentially restrains metabolic capacity under normal conditions of oxidative stress. While antioxidant administration in this population likely induced an increase in muscle blood flow by enhanced scavenging of free radicals and a subsequent increase in nitric oxide (NO) bioavailability [13], it may have also directly improved mitochondrial function [14], thus complicating the interpretation of the findings. Given these apparent conflicting results regarding O_2_ supply and demand in the elderly, circulatory occlusion and the subsequent reactive hyperemia (RH) upon cuff release at the offset of exercise appears to be an interesting alternative experimental model that can, rather simplistically and substantially, locally increase O_2_ supply [15]. Lacking of the limitations of the previous studies, the application of this approach in the elderly would thus allow the assessment of the putative mismatch between peripheral O_2_ supply capacity and intrinsic mitochondrial capacity for ATP synthesis with advancing age.

Therefore, the purpose of this study was to utilize an integrative approach combining vascular and metabolic measurements (^31^P-MRS, NIRS, and Doppler ultrasound) to determine whether, in the elderly, mitochondrial function is limited by O_2_ supply or the intrinsic mitochondrial capacity to synthesize ATP. To this aim, we examined whether a period of brief ischemia during exercise followed by RH would affect limb and capillary blood flow, tissue re-oxygenation and mitochondrial function in old untrained subjects. Based upon the work of Wray et al. [12] in the young and old, utilizing a somewhat different approach, we hypothesized that improved convective O_2_ delivery and capillary blood flow induced by superimposing RH at the offset of exercise would increase tissue O_2_ availability and ultimately result in a higher peak mitochondrial ATP synthesis rate in older subjects.

## RESULTS

### Baseline

Table 1 summarizes tissue oxygenation indices, intracellular metabolite concentrations and pH at rest and at the end of the exercise. Immediately before each exercise bout (i.e. baseline), microvascular oxygenation (tissue oxygenation and deoxyhemoglobin), pH, and phosphorylated compounds ([PCr], [Pi] and [ADP]) were not significantly different between conditions (*P* > 0.05). Baseline mean arterial pressure was not significantly different between conditions (FF: 80 ± 30 mmHg; RH: 82 ± 32 mmHg, *P*>0.05)

**Table 1:** Metabolic and oxygenation indices at rest and the end of plantar flexion exercise in free flow and reactive hyperemia conditions

	Free Flow			Reactive Hyperemia			
*Metabolism*							
*Resting concentrations*							
PCr (mM)	29	±	7	29		5	
Pi (mM)	2.2	±	0.6	1.6		0.6	
ADP (μM)	15	±	10	15		8	
pH	6.99	±	0.02	6.96		0.03	
PDE (mM)	1.5	±	1.3	1.5		1.3	
*End Exercise concentrations*							
PCr (mM)	21	±	6	15	±	4	*
Pi (mM)	8	±	3	13	±	3	*
ADP (μM)	45	±	20	67	±	29	*
pH	7.00	±	0.06	6.94	±	0.06	*
*Microvascular oxygenation*							
*Resting*							
Tissue oxygenation (%)	58	±	11	59		10	
Deoxyhemoglobin (μM)	23	±	8	23		8	
*End Exercise*							
Tissue oxygenation index (%)	50	±	25	39	±	36	*
Deoxyhemoglobin (μM)	25	±	10	34	±	12	*

Data expressed as mean ± SD. Phosphocreatine, PCr; Inorganic phosphate, Pi; ADP; Adenosine diphosphate; Phosphodiester, PDE. * reactive hyperemia significantly different from free flow (P < 0.05).

### Exercise

By the end of exercise, both microvascular oxygenation and the metabolic response differed significantly between free-flow and with circulatory occlusion (P < 0.05, Table 2). Mean arterial pressure was significantly augmented at the end of the exercise with circulatory occlusion (FF: 98 ± 14 mmHg; RH: 109 ± 24 mmHg, P<0.05).

**Table 2:** Phosphocreatine and tissue oxygenation dynamics during the recovery from plantar flexion exercise in free flow and reactive hyperemia conditions

	Free Flow			Reactive Hyperemia			
*PCr offset kinetics and mitochondrial function*							
Amplitude (mM)	9	±	3	15	±	4	*
Time constant (s)	46	±	19	39	±	9	
V_max_ (mM.min^−1^)	21	±	10	29	±	8	*
*Deoxyhemoglobin offset kinetics*							
Time Delay (s)	34	±	46	4	±	5	
Amplitude (%)	3	±	2	11	±	5	*
Tau (s)	43	±	40	22	±	18	
Mean Response Time (s)	77	±	70	26	±	20	
*Tissue oxygenation offset kinetics*							
Time Delay (s)	14	±	15	4	±	6	
Amplitude (%)	9	±	14	24	±	31	*
Tau (s)	67	±	89	19	±	10	
Mean Response Time (s)	81	±	90	22	±	11	

All values are expressed as mean ± SD. Peak rate of oxidative ATP synthesis, V_max_. * reactive hyperemia significantly different from free flow (*P* < 0.05)

### Recovery period

#### Blood Flow and tissue oxygenation

**Figure 1:**
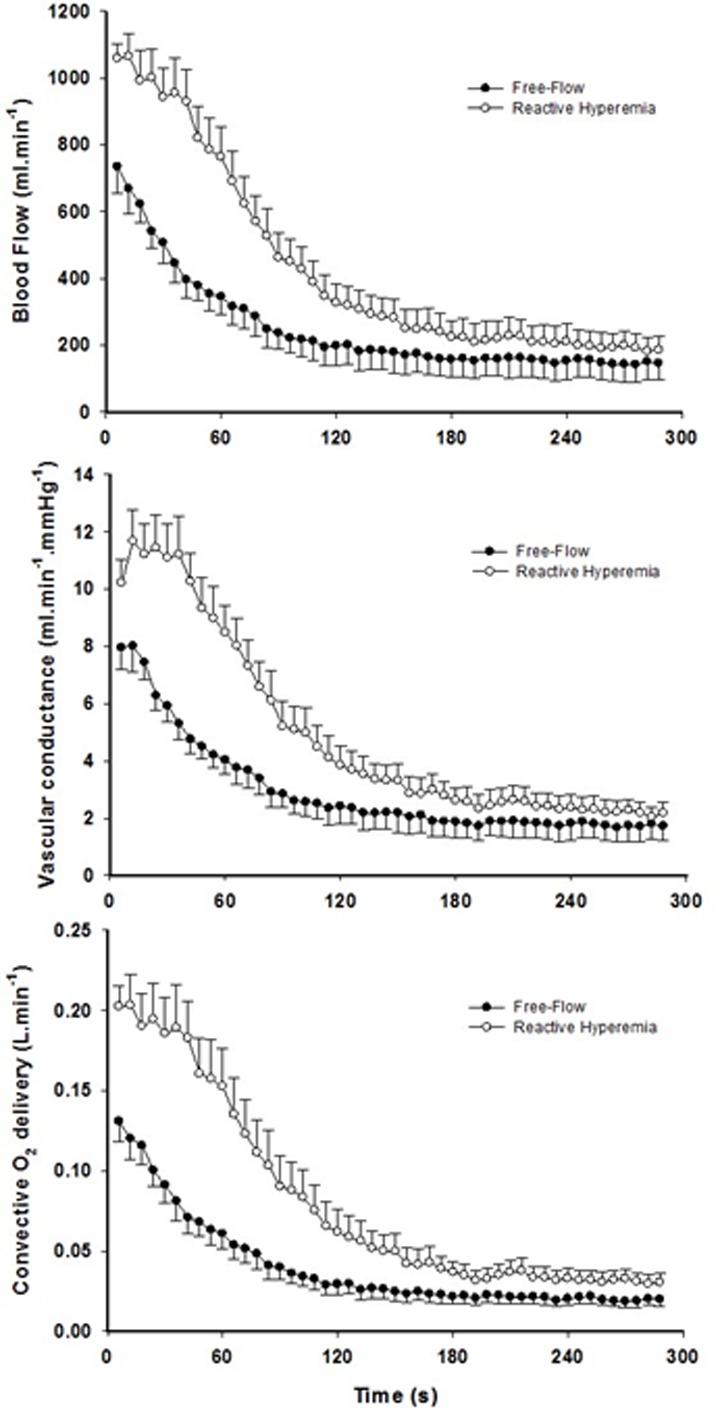
The effect of superimposing reactive hyperemia on the recovery from plantar flexion exercise on the peripheral arterial vasculature and O_2_ delivery. Blood flow (upper panel), vascular conductance (middle panel) and convective O_2_ delivery (lower panel) kinetics in older subjects Area under the curve for blood flow (*P* < 0.01), vascular conductance (*P* < 0.01), and convective O_2_ delivery (*P* < 0.01) were all significantly greater in reactive hyperemia compared to free-flow conditions. Data are presented as mean ± SEM.

**Figure 2:**
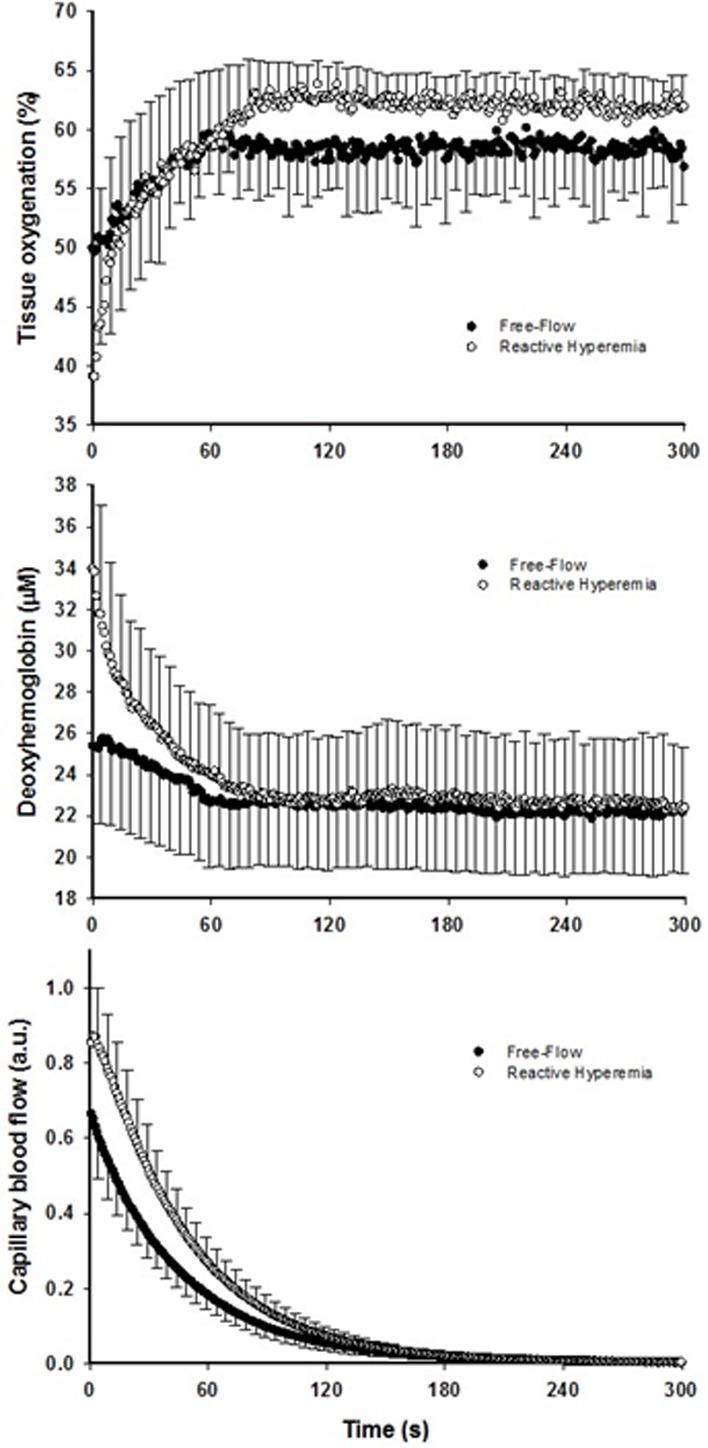
The effect of superimposing reactive hyperemia on the recovery from plantar flexion exercise on the peripheral microcirculation. Tissue oxygenation (upper panel), deoxyhemoglobin (middle panel), and capillary blood flow (bottom panel) kinetics in older subjects Data are presented as mean ± SEM. Capillary blood flow and tissue oxygenation AUC were significantly greater in reactive hyperemia compared to free flow conditions (*P* < 0.05 and *P* < 0.01, respectively).

Mean changes in blood flow, leg vascular conductance and convective O2 delivery dynamics during the recovery period in FF and RH conditions are displayed in Figure 1. Blood flow (FF: 1545 ± 549 ml; RH: 3262 ± 1165 ml; P < 0.01), leg vascular conductance (FF: 21 ± 9 ml.mmHg^−1^; RH: 38 ± 13 ml.mmHg^−1^; P < 0.01) and convective O_2_ delivery (FF: 0.30 ± 0.13 L.mmHg^−1^; RH: 0.64 ± 0.29 L.mmHg^−1^; P < 0.01) AUCs were all significantly greater in RH compared to FF. This augmented blood flow with RH resulted in an increased capillary blood flow (Figure 2) as indicated by the higher AUC in RH compared to FF (P < 0.05).

Although faster, the re-oxygenation kinetics were not significantly different owing to heterogeneous dynamics following the exercise-induced hyperemia (Figure 2, Table 2). However, a greater response in terms of amplitude during RH for both TOI and HHb was detected (P < 0.05). As a consequence, TOI (P < 0.01) AUC was significantly greater in RH compared to FF. Although not reaching significance (P = 0.07), Hbtot was greater upon release of the circulatory occlusion compared to FF (FF: 57 ± 28 μM; RH 64 ± 34 μM).

#### Metabolic offset kinetics assessed with ^31^P-MRS

**Figure 3:**
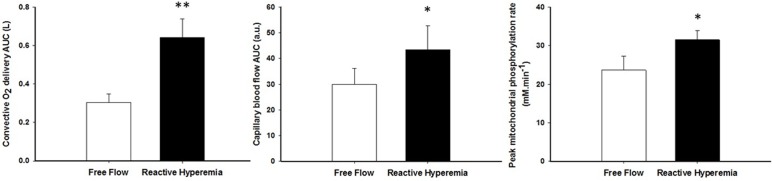
The effect of superimposing reactive hyperemia on the recovery from plantar flexion exercise on convective oxygen delivery (left panel), capillary blood flow (middle panel), and peak mitochondrial phosphorylation rate (V_max_)(right panel) Data are presented as mean ± SEM. * Reactive hyperemia significantly different from free flow condition (*P* < 0.05), ** (*P* < 0.01).

Although shorter, the PCr resynthesis time constant did not reach significance in RH in comparison to FF (P = 0.15, Table 3), whereas the estimated mitochondrial ATP synthesis rate (V_max_) was significantly improved in RH (P < 0.05, Figure 3). The change in V_max_ from RH to FF was neither correlated to changes in pH (r = −0.19, P = 0.56) nor [PCr] (r = −0.35, P = 0.26).

**Table 3:** Subject characteristics

				Normal Range
Sample size	12			
Age (years)	68	±	8	
*Anthropometric characteristics*				
Height (cm)	173	±	9	
Weight (kg)	74	±	12	
BMI (kg/m^2^)	25	±	3	
Lower leg muscle volume (dL)	21	±	4	
Lower leg adipose tissue thickness (mm)	54	±	18	
*Functional characteristics*				
Steps per day	7047	±	2573	
Moderate to vigorous activity (min/day)	36	±	20	
*Blood characteristics*				
Glucose (mg/dl)	90	±	11	(74-106)
Cholesterol (mg/dl)	199	±	42	(118-210)
Triglycerides (mg/dl)	132	±	67	(30-150)
HDL (mg/dl)	49	±	11	(35-72)
LDL (mg/dl)	132	±	67	(0-100)
WBC (K/ul)	5.2	±	1.3	(3.7-9.9)
RBC (M/ul)	5.0	±	0.5	(4.0-5.6)
Haemoglobin (g/dl)	15.2	±	1.7	(12.0-16.1)
Hematocrit (%)	45	±	4	(37.0-47.1)
Neutrophil (K/ul)	3	±	1	(1.9-8.0)
Lymphocyte (K/ul)	1.5	±	0.6	(0.9-5.2)
Monocyte (K/ul)	0.5	±	0.1	(0.16-1.50)

Data expressed as mean ± SD. Body mass index, BMI; high density lipoprotein, HDL; low density lipoprotein, LDL; white blood cells, WBC; red blood cells, RBC; normal range, ().

## DISCUSSION

Utilizing an integrative approach, this study sought to determine whether superimposing reactive hyperemia on the recovery from plantar flexion exercise would positively affect capillary blood flow, tissue re-oxygenation, and ultimately peak mitochondrial respiration rate in old untrained individuals. The three main findings of this study were that the release of brief circulatory occlusion at the offset of plantar flexion exercise in the elderly 1) substantially increased convective O_2_ delivery and muscle capillary blood flow, 2) this resulted in greater tissue re-oxygenation and microcirculatory O_2_ extraction, and 3) substantially improved peak mitochondrial ATP synthesis rate. Together, these findings indicate that an increase in O_2_ availability can enhance mitochondrial function in the plantar flexor muscles of the elderly, thereby revealing a metabolic reserve in skeletal muscle despite advancing age and low level of physical activity.

### Increased convective O_2_ delivery and diffusional conductance for O_2_ after circulatory occlusion

The brief period of ischemia during exercise and the subsequent superimposing of the RH on the typically mono-exponential decay of blood flow post-exercise (FF) enhanced convective O_2_ delivery by 111% (Figures 1 and 3). Likewise, both limb blood flow and leg vascular conductance AUC were substantially augmented (+111% and +77%, respectively, Figure 1). As a result of these changes in bulk limb blood flow, capillary blood flow was significantly enhanced (+45%, Figure 2) as was microvascular re-oxygenation AUC (Figure 2), implying an improvement in local O_2_ availability.

Several mechanisms have been previously suggested to account for this increased muscle O_2_ delivery post-occlusion [15]. Among them, the accumulation of vasoactive metabolites and the subsequent reduction in vascular tone during the circulatory occlusion appear to be the most likely explanations. Accordingly, we observed a greater accumulation of [Pi] and a more pronounced acidosis at the end of exercise in RH in comparison to FF (Table 2). In addition, end-exercise microvascular oxygenation was significantly lower during the ischemic exercise, thus potentially eliciting the release of nitric oxide from the endothelium [16] or erythrocytes in the form of S-nitrosohemoglobin [17], or by augmenting the O_2_ dependent ATP-induced vasodilation in the microcirculation [18, 19]. In addition to increasing bulk blood flow, these local adaptations would also promote an improvement in the regional match between microvascular perfusion and O_2_ utilisation post cuff occlusion thus further increasing the O_2_ availability in the active fibers.

**Figure 4:**
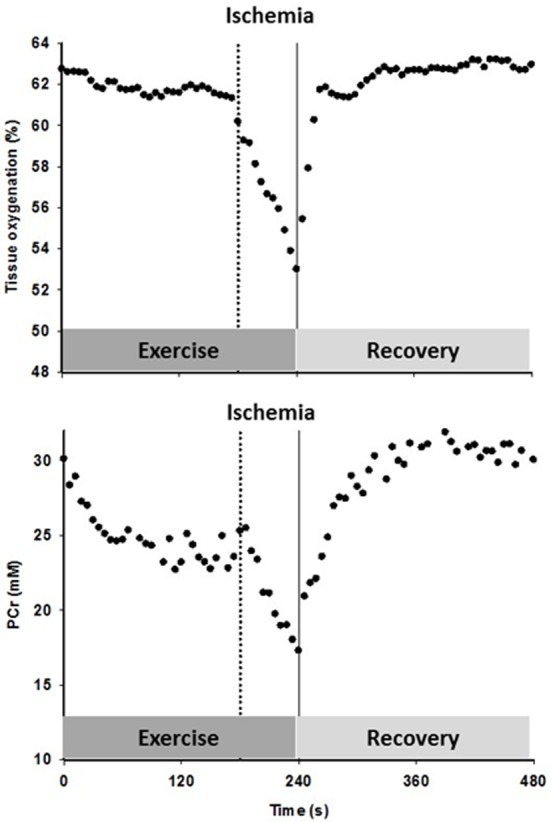
The effect of superimposing reactive hyperemia on the recovery from plantar flexion exercise on the relationship between microvascular partial pressure of O_2_ (PO_2_) and initial post exercise PCr resynthesis rate, an index of O_2_ utilization, in the context of diffusive and convective O_2_ transport Muscle O_2_ utilization was inferred from the initial post exercise PCr resynthesis rate as this process is derived almost exclusively from oxidative phosphorylation [22]. Microvascular PO_2_ was derived from the tissue oxygen index [23], assuming that the near infrared spectroscopy signal mainly originates from hemoglobin [24], and then computed from the O_2_-hemoglobin dissociation curve [25]. Oxygen consumption, VO_2_; blood flow, Q; arterial oxygen content, CaO_2_; venous oxygen content, CvO_2_; diffusional conductance, DO_2_; Mean Capillary PO_2_, PCapO_2_.

In parallel with the large increase in convective O_2_ delivery in RH, it could be postulated that O_2_ diffusional conductance was also improved. Interestingly, the theoretical framework for the interaction between convective and diffusive O_2_ transport, developed by Wagner [20, 21], offers to shed some light on this question. Using a modified version of this approach (Figure 4), it is readily apparent that, in addition to the RH-induced augmentation of convective O_2_ transport, diffusional O_2_ conductance was increased by ∼90% in RH compared to FF. In fact, the increase O_2_ diffusional conductance in RH was similar in magnitude to the increase in convective O_2_ delivery (+111%), indicating that both components of O_2_ transport contributed, almost equally, to the improvement in muscle O_2_ utilization. In the present experimental conditions several mechanisms may have concurrently led to an enhanced capillary surface area available for O_2_ exchange, and thus greater diffusional O_2_ conductance in RH: 1) increased blood flow in originally poorly perfused capillaries (better perfusion-metabolism matching), 2) greater capillary hematocrit, as indicated by an 8% increase in total hemoglobin measured by NIRS, and 3) increased red blood cell velocity and fractional O_2_ extraction, increasing the length of individual capillaries over which blood–tissue O_2_ flux occurs (longitudinal recruitment) [26], as evidenced by both the greater capillary blood flow and deoxyhemoglobin signal (Figure 2).

### Potential age-related blunting of the hemodynamic reserve in the microcirculation

The relative mismatch between the considerable increase in peripheral circulation through large conduit vessels with the RH (+111%), while capillary blood flow was only increased by 45%, is intriguing. By comparison, in a prior study using a similar experimental paradigm, young untrained subjects exhibited parallel improvements in limb and capillary blood flow upon cuff release (+75% and +65% respectively) such that microvascular re-oxygenation was also substantially improved and almost immediately overshoot upon cessation of the exercise in RH [15]. This contrasts to the relatively modest increase in TOI AUC observed in the current study. Together, these observations provide evidence of a hemodynamic reserve in the contracting calf muscle accessible by superimposing reactive hyperemia. However, this appears, at least to some extent, to be attenuated in the microcirculation of elderly untrained individuals.

Conceptually, several potential mechanisms could be responsible for this blunted hemodynamic reserve in the calf microcirculation with age. Although structural limitations and diminished vascular smooth muscle responsiveness may play a role in this finding [27], there is some evidence against this hypothesis. Indeed, skeletal muscle capillary density appears to be relatively well preserved in the lower extremity [28, 29]. Likewise, peak conductance following limb arterial occlusion at rest, an index of arteriolar cross-sectional area [30], is only modestly affected by age in the vasculature of the lower-extremity [27, 31]. Therefore, it is more likely that the relative mismatch between changes in peripheral circulation through large conduit vessels and the microcirculation during superimposed reactive hyperemia stems from an inadequate redistribution of blood flow upon cuff release [32, 33]. In this scenario, a substantial fraction of the enhanced bulk blood flow would be directed toward the non-contracting tissues such that the active regions of the skeletal muscle assessed by NIRS would not benefit from the greater O_2_ delivery as much as in younger adults [15]. This interpretation is supported by previous evidence indicating an age-related impairment in vasomotor control that limits the redistribution of blood flow during muscle contraction in the elderly [34].

The local regulation of muscle blood flow involves a balance between vasodilator and vasoconstrictor influences, both of which have been suggested to be altered with age. There is, indeed, accumulating evidence of an overall increase in plasma free radical concentration with advancing age and a subsequent reduction in NO bioavailability, which has been implicated in the detrimental effect of age on endothelial function [13], movement-induced hyperemia [35, 36], and vasodilatory reserve [37]. Furthermore, the acute administration of an antioxidant cocktail has been documented to enhance post-exercise tissue perfusion in the calf of older individuals [12] thereby providing additional evidence that limited NO bioavailability induced by chronic oxidative stress is a key component in the dysregulation of skeletal muscle blood flow in the elderly.

An increased vascular tone [38, 39], which is a common feature of older sedentary individuals, can also, at least in part, account for the blunted hemodynamic reserve in the calf microcirculation with age. Specifically, an augmented responsiveness to vasoconstrictor substances locally released by the endothelium, such as endothelin-1 [40] and angiotensin II [41] has previously been documented in older individuals. However, it is still equivocal whether functional sympatholysis, the ability to counteract the vasoconstrictor stimuli from the sympathetic nervous system in contracting skeletal muscle, is affected by age in the lower limb [42, 43].

### Evidence of a metabolic reserve in aging skeletal muscle

According to our hypothesis, enhanced O_2_ availability increased peak mitochondrial phosphorylation rate by 36% (Figure 3). Although not significant, the PCr recovery time constant also tended to be faster in RH (P=0.15). This difference is likely related to the contrasting end-exercise muscle milieu in the RH and FF conditions (Table 2), which is well recognized to modulate the exercise PCr offset time constant [44, 45] and confound the interpretation of this index. This inconsistency between the two indices can also be explained by the fact that our sample size was estimated to detect a large effect size on the peak mitochondrial phosphorylation rate. Given the greater variability of the PCr recovery time constant in the present experimental conditions, identifying a significant effect on this index would require to double the sample size to confirm our conclusion with the more robust Vmax parameter.

Overall, our results are consistent with the previously reported faster post-exercise PCr recovery kinetics induced by enhanced tissue perfusion following acute antioxidant administration in elderly subjects [12]. It should, however, be noted that, unlike the present experimental paradigm, antioxidant administration may exert both direct and indirect effects on muscle metabolism as enhanced scavenging of free radicals can induce a subsequent increase in NO bioavailability, which has been documented to improve both muscle blood flow [46, 47] and mitochondrial function [14, 48]. Based upon these considerations, our results provide the first unambiguous evidence of an excess capacity of mitochondrial phosphorylation relative to peripheral O_2_ transport in the calf muscle of untrained elderly subjects.

**Figure 5:**
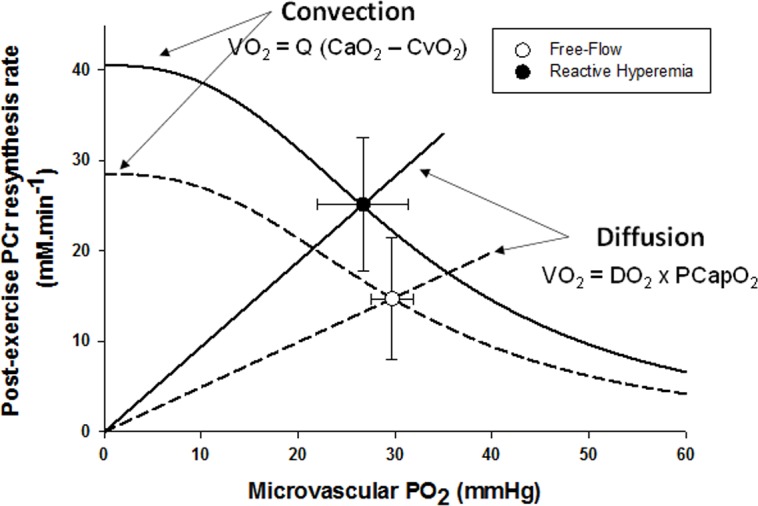
The timeline for the constant-load sub-maximal plantar flexion exercise and recovery, illustrated with an example of the tissue oxygenation and phosphocreatine (PCr) response to the protocol including cuff occlusion during the last min of exercise and the subsequent reoxygenation in an old subject

Interestingly, a prior study from our group using hyperoxia to increase the O_2_ gradient from blood to muscle did not observe such an improvement in mitochondrial function in sedentary older individuals [11]. These findings were interpreted as evidence that mitochondrial function in this population was likely limited by intrinsic mitochondrial capacity rather than O_2_ availability, which appears to be in conflict with the current findings. However, in this prior study, O_2_ availability was altered predominantly by an increase in blood PO_2_ augmenting the O_2_ gradient from blood to mitochondria. Owing to the sigmoidal shape of the O_2_-hemoglobin dissociation curve at high PO_2_ and the already almost fully saturated hemoglobin in normoxia, gains in arterial O_2_ concentration in hyperoxia are therefore rather small (<10%, [20]). In contrast, in the present study, convective O_2_ delivery was dramatically enhanced in RH (+ 111%, Figure 3) and, interestingly, this also resulted in an improvement in diffusional O_2_ conductance of a similar magnitude (+ 90%, Figure 5). This difference may explain the discrepancy between studies.

One could question whether the increase in peak mitochondrial ATP synthesis rate might be the consequence of the confounding effects of differing end-exercise metabolic states on the calculation of Vmax or a direct effect on mitochondrial enzymatic activities. As for the first point, unlike the PCr recovery time constant, Vmax has been repeatedly demonstrated to be independent from the end-exercise metabolic state [49–51]. In agreement with this literature, Vmax was neither correlated to changes in pH nor [PCr] thus ruling out differing end-metabolic states as a confounding factor. As for the second point, several in vivo and in vitro studies suggest that, in conditions of high respiratory rate (ADP-stimulated respiration in isolated mitochondria or during the transition from exercise to rest), intrinsic mitochondrial function is not influenced by differences in metabolic state. For instance, lowering the pH from 7.4 to 6.38 did not affect the maximal ADP-stimulated respiration in isolated mitochondria from the skeletal muscle [52]. Consistent with this in vitro finding and our correlation analyses, the maximum rate of mitochondrial ATP synthesis measured by ^31^P-MRS has been documented to be similar following plantar-flexion exercise at two different durations, despite vastly different pH and metabolite concentrations [50]. Combined with the finding that the maximum rate of mitochondrial ATP synthesis is independent of pH and [PCr] across a wide range of metabolic states (pH ranging from 7.1 to 6.0, and [PCr] from 35 mM to 2 mM, [49, 51], these data suggest that it is very unlikely that the greater V_max_ in RH was the result of an intrinsic improvement in mitochondrial function.

### Perspective and significance

Despite the current evidence of a mitochondrial reserve in the elderly, one should not completely dismiss the potential role of factors downstream from the convective delivery of O_2_ in terms of contributing to the decline in muscle aerobic function with advancing age. Indeed, using an animal preparation, Hepple et al. [53] elegantly illustrated the complex interaction between muscle oxidative capacity and O_2_ delivery which determine maximal O_2_ uptake (VO_2max_) in skeletal muscle. Specifically, a combined reduction in O_2_ delivery and mitochondrial oxidative capacity resulted in a greater impairment in muscle VO_2max_ than either intervention performed independently in young rats. The implication of this finding for the present results is that, even though intrinsic mitochondrial capacity appears to be in relative excess of convective O_2_ transport in the elderly, mitochondrial capacity may still influence muscle VO_2max_ [53]. Therefore, the decline in mitochondrial function [54, 55] and/or density [55] previously suggested by some in vitro reports may still play some role in the impaired ability of aged muscles to use O_2_ during high-intensity exercise.

From a theoretical standpoint, the findings from the present study challenge the classic concept of symmorphosis, which postulates that each component of the O_2_ transport system (heart, blood, capillaries and mitochondria) is matched to demand such that the functional capacity of each step does not limit the maximal overall capacity of the system [56]. Indeed, according to this theory, the peak rate of mitochondrial phosphorylation measured by ^31^P-MRS should not be sensitive to an acute increase in capillary blood flow and O_2_ availability within the skeletal muscle. In addition, despite the well-established impairments in the convective component of O_2_ transport to skeletal muscle with age [1, 2], this does not appear to have resulted in a proportional decrease in mitochondrial function in the elderly, as demonstrated in the present study.

### Methodological consideration

It should be noted that the volume of muscle interrogated by the NIRS was small and localized to the medial gastrocnemius, while, in contrast, the ^31^P-MR signal was detected with a relatively large surface coil, sampling a much greater muscle volume. However, considering that the ^31^P-MR reception sensitivity is greatest close to the coil, the gastrocnemius (lateral and medial) and the soleus contributed to the MR signal in a weighted fashion, with the soleus contributing to a much smaller extent. In addition, this study employed a within-subject comparison, such that ^31^P-MRS and NIRS signals originated, for each method, from the same muscle volume in both conditions. Therefore, while acknowledging the potential influence of these differing measurement approaches, it is reasonable to contend that our qualitative estimate of capillary blood flow was not greatly confounded by sampling volume differences between techniques.

## CONCLUSION

In summary, using an integrative approach, this study has revealed a hemodynamic reserve in the contracting calf muscle accessible under conditions of superimposed reactive hyperemia, which appears, to some extent, to be blunted in the microcirculation with advancing age. In addition, this study provides the first direct evidence of an excess capacity of mitochondrial respiration relative to peripheral O_2_ transport capacity in the calf muscle of untrained elderly

## MATERIALS AND METHODS

### Subjects

Following the attainment of informed consent, 12 healthy sedentary elderly subjects (10 men and 2 women), participated in this study (Table 3). The subjects were recruited based upon no evidence of regular physical activity above that required for activities of daily living (assessed by both questionnaire and accelerometry), and being greater than 60 years of age. All subjects were non-smokers, free of diabetes, and known cardiovascular, peripheral vascular, neuromuscular, or pulmonary disease. Additionally, none of the subjects were taking statins or other medications recognized to affect muscle or vascular function. Women taking hormone replacement therapy were excluded from the study. The study was approved by the Human Research Protection Programs of both the University of Utah and the Salt Lake City VA Medical Center.

### Exercise protocol

After familiarization with the equipment, individual maximum work rate (WR_max_) was determined by performing incremental dynamic plantar flexion exercise until exhaustion. On a separate day, subjects performed constant-load sub-maximal plantar flexion at ∼40% of WR_max_ (frequency of 1 Hz) in the whole body MRI system (TimTrio, 2.9T Siemens Medical Systems, Erlangen, Germany) under conditions of FF and then RH, as PCr recovery kinetics are unaffected by prior exercise [50]. Specifically, after 2 min of rest, subjects exercised for 4 min followed by 5 min of recovery. In RH, min 3-4 was performed under ischemic conditions induced by a cuff occlusion (Figure 5). A blood pressure cuff, placed distal to the knee, was rapidly inflated to 250 mmHg to occlude the popliteal artery. In a subset of 9 subjects, this protocol was repeated on a separate day in order to measure limb and capillary blood flow as well as tissue oxygenation using Doppler ultrasound imaging and NIRS, respectively. Prior to initiation of this protocol, blood samples were collected to perform a complete blood cell count analysis.

### ^31^P MRS

^31^P-MRS was performed using a clinical 2.9T MRI system (Tim-Trio, Siemens Medical Solutions, Erlangen, Germany) operating at 49.9 MHz for ^31^P resonance. ^31^P MRS data were acquired with a dual tuned ^31^P-^1^H surface coil with linear polarization (Rapid biomedical GmbH, Rimpar, Germany) positioned under the calf at its maximum diameter. The ^31^P single-loop coil diameter was 125 mm surrounding a 110 mm ^1^H coil loop. The centering of the coil around the leg was confirmed by T_1_ weighted ^1^H localizing images and the coil was repositioned if the majority of the gastrocnemius muscle was not within this range. For all subjects a similar ratio between the volumes of gastrocnemius / soleus muscles was maintained within the coil. After a three-plane scout proton image, advanced localized volume shimming was performed. Before each experiment, two fully relaxed spectra were acquired at rest with 3 averages per spectrum and a repetition time of 30 s. Then, MRS data acquisition was performed throughout the rest-exercise-recovery protocol using a FID (free-induction-decay) pulse sequence with a 2.56 ms adiabatic-half-passage excitation radiofrequency pulse and the following parameters: repetition time = 2 s; receiver bandwidth = 5 kHz; 1024 data points; and 3 averages per spectrum). Saturation factors were quantified by the comparison between fully relaxed (TR = 30 s) and partially relaxed spectra (TR = 2s).

As previously described [57], relative concentrations of PCr, inorganic phosphate Pi, and ATP were obtained by a time-domain fitting routine using the Advanced Method for Accurate, Robust and Efficient Spectral (AMARES) fitting algorithm [58] incorporated into the CSIAPO software [49]. Intracellular pH was calculated from the chemical shift difference between the Pi and PCr signals. The free cytosolic [ADP] was calculated from [PCr] and pH using the creatine kinase equilibrium constant (K_CK_ = 1.66 × 10^9^ M^−1^) and assuming that phosphocreatine represents 85% of total creatine content [59]. The resting concentrations were calculated from the average peak areas of the three relaxed spectra (TR =30 s; N = 3) recorded at rest, assuming an 8.2 mM ATP concentration. When Pi splitting was evident, the pH corresponding to each Pi pool was calculated separately as pH_1_ and pH_2_ on the basis of the chemical shift of each peak relative to PCr. The overall muscle pH was then calculated as pH = pH_1_ (area Pi_1_/total Pi area) + pH_2_ (area Pi_2_/total Pi area).

### Popliteal blood flow

Measurements of popliteal artery blood velocity and vessel diameter were performed in the popliteal fossa of the exercising leg proximal to the branching of the medial inferior genicular artery with a Logic 7 Doppler ultrasound system (General Electric Medical Systems, Milwaukee, WI). The ultrasound system was equipped with a linear transducer operating at an imaging frequency of 10 MHz. Vessel diameter was determined at a perpendicular angle along the central axis of the scanned area. Blood velocity was measured using the same transducer with a frequency of 5 MHz. All blood velocity measurements were obtained with the probes appropriately positioned to maintain an insonation angle of 60° or less. The sample volume was maximized according to vessel size and was centered within the vessel. Arterial diameter was measured off-line every 12 s using automated edge-detection software (Medical Imaging Applications, Coralville, IA), and mean velocity (Vmean) (angle corrected, and intensity-weighted area under the curve) was automatically calculated beat by beat (Logic 7). Using arterial diameter and Vmean, blood flow in the popliteal artery was calculated as blood flow = Vmean · π (vessel diameter/2)^2^ · 60, where blood flow is in milliliters per minute. Mean arterial pressure (MAP), heart rate, stroke volume, and cardiac output were determined with a Finometer (Finapres Medical Systems, Amsterdam, The Netherlands). Leg vascular conductance was then calculated as popliteal artery blood flow divided by MAP. Arterial O_2_ content (CaO_2_) was calculated as the sum of bound O_2_ (0.0134 · Hb · SaO_2_) and dissolved O_2_ (0.000031 · PO_2_) assuming a constant SaO_2_ = 94 % and PO_2_ = 70.8 mmHg, based upon typical values and a normal Hb association curve [60]. O_2_ delivery was then calculated as the product of CaO_2_ and popliteal artery blood flow.

### Microvascular oxygenation and capillary blood flow

Microvascular oxygenation was assessed using the NIRS technique, which provides continuous, non-invasive measurements of oxygenated (HbO_2_), deoxygenated (HHb) and total (Hbtot) haemoglobin levels as well as a tissue oxygenation index (TOI, i.e. HbO_2_/Hbtot). Due to identical spectral characteristics, haemoglobin and myoglobin are not separated using NIRS. However, the signal is usually considered as being derived mainly from Hb [24]. In the present study, changes in microvascular oxygenation of the right *gastrocnemius* muscle were continuously monitored at 2 Hz using a near infrared frequency resolved spectroscopy oximeter (Oxiplex TS, ISS Inc., Illinois USA). The probe was positioned at the level of the largest circumference of the medial *gastrocnemius* and secured with Velcro straps and bi-adhesive tape. NIRS uses intensity-modulated light and the probe consisted of 8 infrared light sources (4 emitting at 690 nm and 4 emitting at 830 nm) and one detection channel (interoptode distance = 1.5 to 4.5 cm) including a selected light detector (photomultiplier tube), thus providing a measurement of absorption and the scattering coefficient of the tissues. Measurement of adipose tissue thickness under the NIRS sample site was performed with a Logic 7 Doppler ultrasound system (General Electric Medical Systems, Milwaukee, WI).

As previously described [15], the estimated capillary blood flow response following the offset of exercise was calculated from a modified version of the method proposed by Ferreira *et al.* [61, 62] using the kinetics of muscle O_2_ consumption and the HHb data. Specifically, the PCr resynthesis rate, measured by ^31^P-MRS, which is derived almost exclusively from oxidative phosphorylation [22], was used as an index of muscle O_2_ consumption. Then, as the HHb response determined by NIRS is considered to reflect muscle capillary O_2_ extraction (i.e., CaO_2_-CvO_2_) [24], and based upon the Fick equation, the temporal characteristics of capillary blood flow were estimated using the PCr resynthesis rate to HHb ratio.

### Data analysis

The PCr, HHb, and TOI recovery kinetics were determined by fitting the time-dependent changes during the recovery period to a mono-exponential curve described by the following equation:

$$Y\;(t)=Y_{\text{end}}+Y_{\mathrm{res}}\;(1-e^{-(t-\mathrm{TD}/\tau )})$$ (1)

where Y_end_ is the level of [PCr], HHb, or TOI measured at end-of-exercise and Y_res_ refers to the amount of PCr resynthesized or the resaturation during the recovery. Unlike TOI or HHb, there is no time delay (TD) in the resynthesis of PCr and therefore TD was fixed to 0 for PCr kinetics. Then, the initial rate of PCr resynthesis from ^31^P-MRS (Vi_PCr_) was calculated from thederivative of equation (1) at time zero:

$${\mathrm{Vi}}_{P}{}_{\mathrm{Cr}}=k\cdot \Delta [\mathrm{PCr}]$$ (2)

in which Δ [PCr], represents the amount of PCr resynthesized during the recovery and the rate constant k = 1/τ [63].

Then, peak rate of oxidative ATP synthesis from ^31^P-MRS (V_max_ in mM.min^−1^) was calculated using the initial rate of PCr synthesis (Vi_PCr_) during the recovery period and [ADP] obtained at the end of exercise as previously described [64]:

$$V_{\max}={\mathrm{Vi}}_{\mathrm{PCr}}(1+(K_{m}/{\left[\mathrm{ADP}\right]}_{\text{end}}{}^{2.2}))$$ (3)

in which K_m_ (the [ADP] at half the highest oxidation rate) is 30 μM in skeletal muscle [63].

Model variables were determined with an iterative process by minimizing the sum of squared residuals (RSS) between the fitted function and the observed values. Goodness of fit was assessed by visual inspection of the residual plot and the frequency plot distribution of the residuals, Chi square values, and the coefficient of determination (r^2^), which was calculated as follows [65]:

$$r^{2}=1-\;({\mathrm{SS}}_{\mathrm{reg}}{/\mathrm{SS}}_{\mathrm{tot}})$$ (4)

with SSreg, the sum of squares of the residuals from the fit and SStot, and the sum of squares of the residuals from the mean. In a subset of subjects demonstrating the greatest drop in pH (n=4), we determined that a single exponential function rather than a double exponential function [66] provided the best fit of PCr recovery kinetics.

### Statistical Analysis

The assessment of differences between FF and RH was performed with either paired t-tests or nonparametric Wilcoxon tests, where appropriate (Statsoft, version 5.5; Statistica, Tulsa, Oklahoma). Popliteal blood flow, leg vascular conductance, convective O_2_ delivery, capillary blood flow and TOI cumulative area under the curve (AUC) was calculated as the summed sec-by-sec response during the first 180 s of recovery and used to identify how differences over time were affected by the treatment. Statistical significance was accepted at *P* < 0.05. Results are presented as mean ± SD in tables and mean ± SEM in figures for clarity.
